# Pharmacological inhibition of USP18 improves antibacterial responses and the intracellular control of *Mycobacterium tuberculosis* in macrophages

**DOI:** 10.3389/fimmu.2026.1739628

**Published:** 2026-03-04

**Authors:** Qiao Zhang, Zhen Gong, Robert Schnell, Yi Zhong, Paul P. Geurink, Volker M. Lauschke, Jianping Xie, Stefano Gastaldello, Martin E. Rottenberg

**Affiliations:** 1 Department of Microbiology, Tumor and Cell Biology, Karolinska Institutet, Stockholm, Sweden; 2 School of Life Science, Southwest University, Chongqing, China; 3 Department of Clinical Laboratory, The Second Affiliated Hospital, Anhui Medical University, Hefei, China; 4 Department of Neuroscience, Karolinska Institutet, Stockholm, Sweden; 5 Department of Molecular Neurosciences, Center for Brain Research, Medical University of Vienna, Vienna, Austria; 6 Department of Physiology and Pharmacology, Karolinska Institutet, Stockholm, Sweden; 7 Pharmaceutical Informatics Institute, College of Pharmaceutical Sciences, Zhejiang University, Hangzhou, Zhejiang, China; 8 Department of Cell and Chemical Biology, Leiden University Medical Center, Leiden, Netherlands; 9 Dr Margarete Fischer-Bosch Institute of Clinical Pharmacology, Stuttgart, Germany; 10 Department of Pharmacy, The Second Xiangya Hospital, Central South University, Changsha, China

**Keywords:** ISG15, ISGylation, macrophage, *Mycobacterium tuberculosis*, type I interferon, USP18

## Abstract

**Introduction:**

Type I interferon (IFN-I) responses correlate with the severity of Tuberculosis. ISG15, a ubiquitin-like protein, regulates immune responses intracellularly by protein posttranslational modification (ISGylation) or extracellularly, in a cytokine-like manner. The ubiquitin specific protease USP18 deconjugates ISG15 from target proteins and is a major negative regulator of IFN-I signaling.

**Methods:**

Here we show that *M. tuberculosis* infection induces ISGylation associated transcripts in murine bone marrow-derived macrophages (BMM). The expression of these transcripts was dependent on IFN-I signaling and was further enhanced by supplementation of IFN-β, but not with IFN-γ or IL-1β. While stimulation with IFN-β impaired the intracellular control of *M. tuberculosis* in BMM, the bacterial growth was not altered by addition of extracellular ISG15. Treatment with two USP18 inhibitors, increased the ISGylation in uninfected and *M. tuberculosis*-infected BMM.

**Results:**

The inhibition of USP18 enhanced both IFN-I responses and antimicrobial responses in BMM and increased NF-κB-activation and IL-1β expression. Moreover, the USP18 inhibitors improved the intracellular macrophage control of *M. tuberculosis* in an IFN-I independent manner. Instead, the addition of IFN-β to the BMM cultures at least partially reversed the bacterial control conferred by the USP18 inhibition.

**Discussion:**

Our results suggest that the pharmacological inhibition of USP18 can be further explored as a therapeutic tool in the control of tuberculosis.

## 1 Introduction

Tuberculosis (TB), caused by the infection with *Mycobacterium tuberculosis (M. tuberculosis)*, remains a leading public health problem worldwide. The worldwide incidence of TB is approximately 10 million new cases and 1.4 million deaths annually (https://www.who.int/news-room/fact-sheets/detail/tuberculosis). The rate of progression from infection to disease is highly variable. While approximately 90% of infected individuals never develop clinical disease, in 5-10% of cases *M. tuberculosis* causes an extensive lung damage and efficient airborne transmission of bacteria (1).

Macrophages are both the major host cell niche where *M. tuberculosis* survives and proliferates, and the cell where the bacilli can be controlled or eliminated. A host counters mycobacterial infections primarily via IFN-γ-mediated macrophage activation (2, 3). In contrast, the type I IFNs family (IFN-I) exacerbates clinical and experimental TB pathogenesis (4–6). IFN-I inhibits the production of the protective cytokine IL-1β by hampering inflammasome activation, and by inducing IL-10, which further reduced IL-1 and IFN-γ gene transcription (7), as well as responsiveness to IFNγ, leading to an initial loss of bacterial control. This action is amplified by neutrophil and plasmacytoid dendritic cells (PDC) activation (5). Although high levels of IFN-I have negative effects in TB, tonic IFN-I signaling or low IFN-I levels may exert beneficial effects by priming host-protective responses (8).

IFN-I mediates the production of numerous innate immune genes, including ISG15, a ubiquitin-like intracellular protein modifier and other molecules involved in the control of ISG15 production. ISG15 expression is upregulated in response to cellular stress caused by bacterial and viral infections (9).

The covalent modification of proteins by ISG15, termed ISGylation, occurs in more than 100 intracellular proteins. ISG15 is synthesized as an inactive precursor the C-terminal peptide cap needs to be removed, to expose the C-terminal glycine required for target binding (10). ISG15 is covalently conjugated onto target proteins via an enzymatic cascade, although the fate of these modified proteins is still largely unknown. However, protein ISGylation has been shown to disrupt the replication of several viruses (9, 11). Unlike ubiquitylation, ISGylation does not directly label the target proteins for proteosomal degradation. In addition to its intracellular conjugation, extracellular ISG15 has been proposed to function as a cytokine with several immunomodulatory activities (12).

The ISG15-binding motif is recognized by the ubiquitin-specific protease 18 (USP18), which removes ISG15 from the conjugated proteins (13). In contrast to other members of the USP family, USP18 shows no reactivity toward ubiquitin but specifically deconjugates ISG15 (14, 15).

All type I IFNs bind to a common cell-surface receptor, known as the type I IFN receptor, which is composed of two subunits, IFNAR1 and IFNAR2. These subunits are associated with the Janus activated kinases (JAKs) tyrosine kinase 2 and JAK1, respectively. Activation of the JAKs that are associated with the type I IFN receptor results in tyrosine phosphorylation of STAT2 and STAT1, leading to the formation of STAT1–STAT2–IRF9 trimers that translocate to the nucleus and binding to interferon-stimulated response elements (ISREs) in the DNA to initiate gene transcription (16). Mutational studies have shown that USP18 binds to the intracellular region of IFN-I receptor subunit IFNAR2 outcompeting the downstream kinase JAK1 and thereby abrogating IFN-I-signaling independent of the peptidase activity (17). Patients with USP18 deficiency showed a type I interferonopathy and predisposition to mycobacterial disease (18, 19). ISG15 interacts with USP18, protecting it from ubiquitylation and subsequent proteasomal degradation. In ISG15-deficient patients USP18 is thus degraded, resulting in a prolonged IFN-I signaling causing a type I interferonopathy and enhanced susceptibility to mycobacterial infection (20–22).

Here, we analyzed the immunological features of ISG15 and USP18 as intracellular regulators of the outcome of *M. tuberculosis* infection in macrophages. Our studies confirm that USP18 modulates IFN-I responses by reducing ISGylation. Moreover, we show that USP18 impairs the intracellular control of *M. tuberculosis* intracellular growth in macrophages through a mechanism that is independent of IFN-I signaling.

## 2 Materials and methods

### 2.1 Ethics

Mice were housed in accordance with the directives and guidelines of the Swedish Board of Agriculture, the Swedish Animal Protection Agency, and the Karolinska Institutet (djurskyddslagen 1988:534; djurskyddsförordningen 1988:539; djurskyddsmyndigheten DFS 2004:4). The study was performed under approval of the Stockholm North Ethical Committee on Animal Experiments permit number 1374–2020 and 2476-2025.

### 2.2 Generation of bone marrow-derived macrophages

Bone marrow cells were flushed from tibia and femurs with PBS, filtered through a 70μm cell strainer, resuspended in DMEM supplemented with 10% FCS and 30% L929 cell-conditioned medium (as a source of macrophage-colony stimulating factor) and incubated for 6 days at 37°C, 5% CO_2_. Bone marrow-derived macrophage (BMM) cultures were then washed with PBS, detached with 1%trypsin, and 5x10^5^ cells were seeded per well in 24-well plates. BMM were further incubated for 24 h at 37°C before infections or treatment with diverse compounds. BMM were F4/80+, CD11b+, CD11c- and SiglecF- (23).

### 2.3 Infection of BMM with *M. tuberculosis*

*M. tuberculosis* Harlingen or H37Rv carrying the green fluorescent protein (GFP)-encoding pFPV2 plasmid were grown in Middlebrook 7H9 (Difco, Detroit, MI) supplemented with albumin, dextrose and catalase and quantified by densitometry. BMM were infected with sonicated bacteria at a multiplicity of infection (MOI) of 2 unless otherwise indicated. After 4 h, cells were washed twice with PBS to remove extracellular bacteria and further incubated for 1 to 5 days. The infected BMM were then detached using trypsin-EDTA at different times after infection, incubated with live/dead stain (LIVE/DEAD™ Fixable Yellow Dead Cell Stain, Invitrogen), washed with FACS buffer (PBS containing 0.5% FCS and 0.5 mM EDTA] and fixed with 4% formaldehyde (Sigma-Aldrich) at room temperature for 10 min. Data were acquired on a Sony ID7000 spectral cytometer and analyzed with FlowJo software (Tree star Inc., Ashland, OR). Cells within the mononuclear gate were ≥ 95% viable, and ≥ 95% of live cells are CD11b+ F4/80+. This procedure allowed the exclusion of *Mtb* associated with dead cells and enable determination of the frequency of infected cells and the relative levels of intracellular bacteria (23).

### 2.4 Small interfering RNA gene silencing

siRNA transfections were performed as previously described (24), using sequences listed in the **Supplementary Table 2**. BMM were plated at 1 × 10^6^ cells per ml in 24-well plates overnight. On the day of transfection, the media was replaced with 450 μl DMEM without penicillin/streptomycin or L-929 cell supernatants. For each target gene, two tubes were prepared. Optimem (25 μl per well) was added to each tube. Lipofectamine RNAimax (2.5 μl per well; Invitrogen) was added to one tube and siRNA (50 nM per well, unless otherwise indicated) was added to the second tube. The siRNA was then combined with the vial with RNAimax, mixed well by pipetting and incubated for 15 min. Then, 50 μl of the mix was incubated with the BMM cell culture. Twenty-four hours after transfection, cells were infected or incubated with different molecules.

### 2.5 Real time PCR

Total RNA was extracted from lung samples or culture cells using Trizol (Sigma Aldrich) and cDNA was obtained by reverse transcription. Transcripts were quantified by real time PCR as described (25). Transcripts were quantified using *hprt* as a control house-keeping gene to calculate the ΔCt values for individual samples. The relative number of transcripts was calculated using the 2^-(ΔΔCt)^ method. The primer sequences used are listed in **Supplementary Table 1**. These values were then used to calculate the relative expression of mRNA in the different conditions (infection and/or treatment) used in tissues and cells.

### 2.6 IL-1β measurement in culture supernatants

The concentration of IL-1β in BMM supernatants were quantified by ELISA, according to the manufacturer’s instructions (BioLegend ELISA MAX™ Standard Set Mouse IL-1β).

### 2.7 RNA sequencing

RNA was extracted from BMM, infected or not with *M. tuberculosis and* treated with the USP18 inhibitor 4 h before infection. RNA was isolated using the miRNeasy micro kit (QIAGEN, Hilden, Germany) according to the manufacturer’s instructions and processed for sequencing at bioinformatics and expression analysis at Novogene Biotech Co (Cambridge, UK). The RNA quality was assessed on a 2200 TapeStation Instrument (Agilent, Santa Clara, CA). PolyA RNA selection was performed using the Illumina TruSeq RNA Sample Preparation Kit according to the manufacturer’s protocol. RNA-seq libraries were prepared and sequenced on the Illumina HiSeq 2000 platform. All the FASTQ files that passed QC were quantified by Salmon (26) using GRCm39 as the reference transcriptome. Differential gene expression analysis was performed with R (version 4.3.3) using the Limma package (27). Genes with adjusted p-value less than 0.05 were and fold change larger than 2 were identified as differentially expressed. All the FASTQ files that passed QC were quantified by Salmon using GRCm39 as the reference transcriptome (26). Differential gene expression analysis was performed with R (version 4.3.3) using the Limma package (27). Genes with adjusted p-value less than 0.05 were and fold change larger than 2 were identified as differentially expressed genes (DEGs). Gene Ontology enrichment analysis (Biological Process, Molecular Functions) or KEGG pathway enrichment was performed with WebGestalt (http://www.webgestalt.org) using default parameters. The raw and processed RNA sequencing data can be found at Gene Expression Omnibus (GEO) repository.

### 2.8 Small molecule inhibitors of USP18

The selection and development of the small molecular inhibitors of USP18 used 2K04 and BB7 have been recently described (28). Briefly, 2K04 was selected from a chemical library of candidate DUBs screened for the inhibition of the USP18 biochemical activity. BB7 is an optimized inhibitor derived by medicinal chemistry and NC is a stereoisomer of BB7 that shows no USP18 inhibition. The inhibitors formed covalent interactions with USP18, and were cell penetrating. The compounds demonstrated an exceptional specificity for murine USP18 across 41 deubiquitinases including 22 USPs (Ubiquitin Specific Proteases) from the related USP family of DUBs (28). Both compounds inhibited mUSP18 enzymatic activity with IC_50_ at the nanomolar range and inhibited the engagement of cellular USP18 with ISG15 at 1 μM. No BMM toxicity was observed at the concentrations used in our study.

### 2.9 Western blot

BMMs were lysed in RIPA buffer (25mM Tris-Cl pH 7.5, 50 mM NaCl, 0.5% NP40, 0.1% SDS, 0.5% DOC, 1mM DTT, 20mM NEM supplied with fresh Protease and Phosphatase inhibitors). A syringe with 29G needle was used to mechanically disrupt genomic DNA. Lysates were clarified at 10000 g for 15 min at 4°C. Protein concentration (DC Protein Assay Kit, Bio-Rad) was determined on the clear supernatants. Desired concentrations of cell lysates were denatured for 10 min at 95°C in loading buffer (NuPage 4X, Reducing Agent 10X, Invitrogen, Carlsbad, CA) and loaded in acrylamide Bis-Tris 4-12% gradient gels (Invitrogen, Carlsbad, CA). After transfer onto PVDF membranes (Merck Millipore, Bedford, MA) for 60 min at 0.34 A, the filters were blocked in TBS-T solution (50 mM Tris-Cl, 150 mM NaCl, 0.1% Tween-20 and 5% non-fat milk, pH 7,6), and incubated with specific primary antibodies (polyclonal rabbit anti-ISG15, monoclonal rabbit anti-phospho-IκBα pSer32, monoclonal rabbit anti-total IκBα), overnight at 4°C, following an incubation with the appropriate horseradish peroxidase-conjugated secondary antibodies for 1 h at room temperature. Proteins were visualized by chemiluminescence (ECL, GE Healthcare, Uppsala, Sweden), detected by the ChemiDoc MP Imaging System (Bio-Rad Laboratory, CA), and chemiluminescence intensity was analyzed only for non-saturated bands using the corresponding imaging analysis software, ImageJ version 5.0.

### 2.10 Recombinant mature and capped ISG15 production

The coding sequences for the full-length and the cap free (removing the C-terminal hexapeptide cap) mouse ISG15 protein (Uniprot Acc. Nr Q64339) were obtained as synthetic genes (Thermo Fisher, GeneArt). The coding sequences were cloned in the pNIC28Bsa4 expression vector (GenBank Acc. Nr. EF198106). The expression constructs contain an N-terminal hexahistidine tag and a tobacco etch virus (TEV) protease recognition site for affinity tag removal; the resulting products carry one extra N-terminal serine residue. The His_6_-tagged proteins were expressed in *E. coli* BL21(DE3). The cells were harvested by centrifugation, resuspended in 25 mM Tris-HCl, 300 mM NaCl, 10 mM imidazole (pH 8.0), and lysed by 40 µg/mL lysozyme, 6 µg/mL DNase-I, and sonication. The clarified lysates were loaded onto a 0.5 mL Ni-NTA column (Thermo Scientific). The His_6_-tagged proteins were eluted by an increasing imidazole gradient.

The imidazole was removed by a PD10 desalting column (Cytiva). The protein preparations were treated with TEV protease for affinity tag removal. The His_6_-tagged TEV protease and un-cleaved proteins were removed using a Ni-NTA column. The tag-free proteins were concentrated using the Amicon concentrator device (Merck) with 3 kDa molecular weight cut-off and loaded on size exclusion chromatography column (Superdex-75, GE Healthcare). The mouse ISG15-capped (immature) was made available at 545 µM concentration, and that ISG15-uncapped (mature) at 1453 µM. The protein preps were aliquoted and stored at –80°C until use. The recombinant mouse ISG15 protein preparations were analyzed in SDS-PAGE and the by mass-spectrometry to control purity and identity (**Supplementary Figure 4**). The sequence coverage of the tryptic peptides from the preparation was 98% for the capped construct and 94,8% for the uncapped product, strongly indicating proper biological functions.

### 2.11 Statistical analysis

All *in vitro* assays were performed at least twice, each time using biological triplicates in each assay. Differences in transcript and protein levels and % infected cells were analyzed by unpaired Student’s *t*-test considering unequal variances (Welch’s test) or by one-way ANOVA using the Welch’s adjustment when comparing three or more groups. When more than two parameters (i.e infection and treatments) were analyzed a two-way ANOVA was applied. Statistical analyses were performed in Prism (GraphPad, La Jolla, CA).

## 3 Results

### 3.1 IFN-βincreases the expression of inflammatory and ISGylation-related genes in *M. tuberculosis*-infected BMM

**Figure 1:**
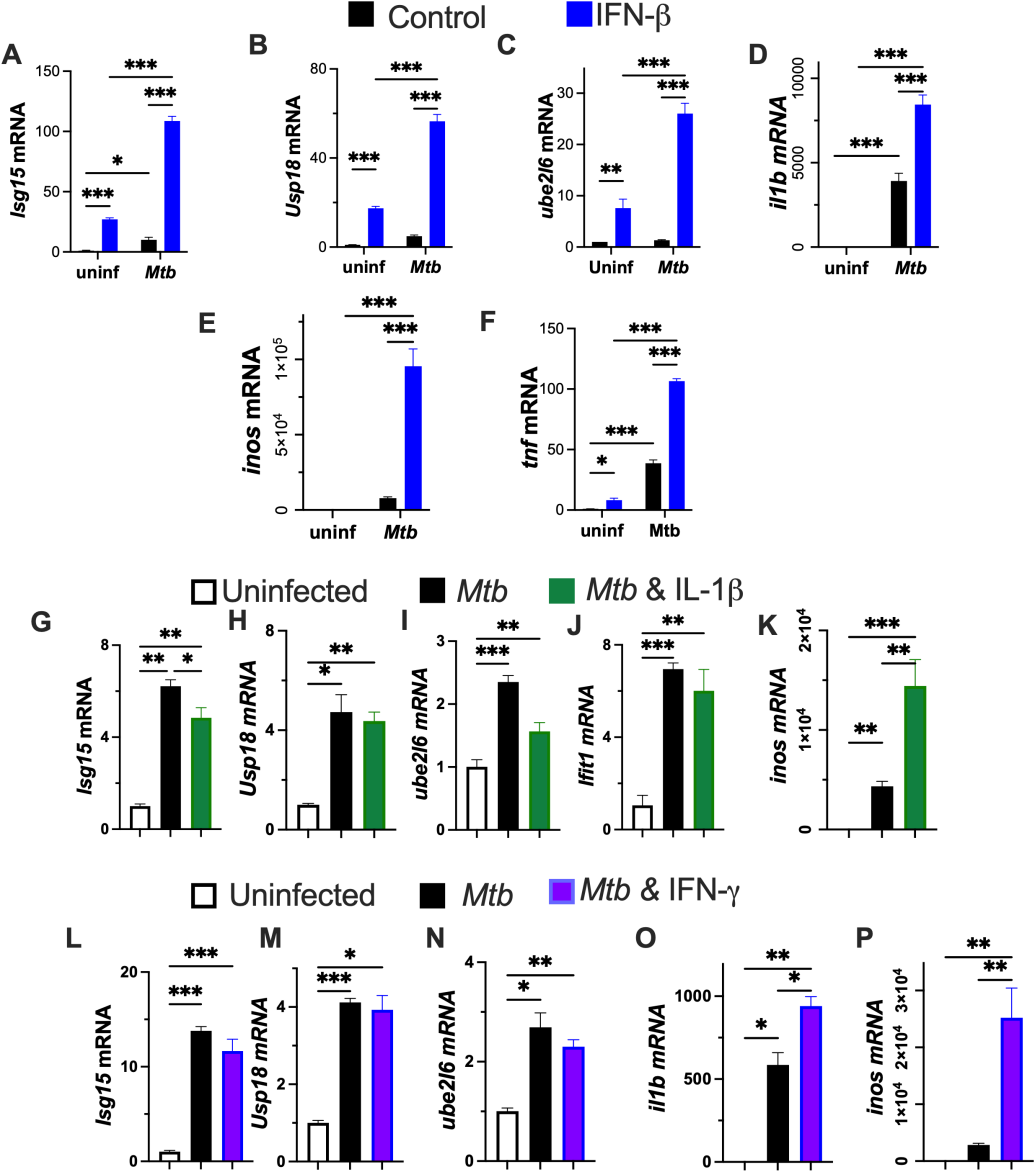
IFN-β increases the expression ISGylation pathway and inflammatory genes in *M. tuberculosis* infected BMM. **(A–F)** BMM were incubated with 5 ng/ml IFN-β later and infected with *Mtb* 24 h after treatment. Total RNA was isolated 24h after infection and the *isg15, usp18*, *il1b*, *inos and tnf* mRNA levels were determined by RT-PCR. **(G–K)** BMM were incubated with 1 ng/ml IL-1β and infected with *M. tuberculosis* 24h after stimulation. Total RNA was isolated 24h after infection and the *isg15, usp18, ube2l6*, *IL-1β* and *inos* mRNA levels were determined by RT-PCR. **(L–P)** BMM were incubated with 1200 U/ml recombinant mouse IFN-γ and infected with *Mtb* 24h after treatment. Total RNA was isolated 24h after infection and *isg15, ube2l6, usp18*, *il1b* and *inos* mRNA levels were determined by RT-PCR. **(A–P)** The mean ± SEM of triplicate independent cultures is depicted. **(A–F)** Differences between groups are significant (* p ≤ 0.05; **p ≤ 0.01 and ***p ≤ 0.001 two-way ANOVA test). **(G–P)** Differences are significant at *p ≤ 0.05, **p ≤ 0.01 and ***p ≤ 0.001, one-way ANOVA.

Type I IFN responses, including several regulators of ISGylation, are increased in TB patients as well as in individuals who progress to active disease (4). In addition to being strongly induced by IFN-I, ISG15 has also been shown to be upregulated by viral and bacterial infections, LPS and retinoic acid (29–31). Therefore, we asked whether *M. tuberculosis* infection influences ISGylation in bone marrow-derived macrophages (BMM). Notably, upon infection, BMM showed a clear upregulation of the expression genes involved in the ISGylation pathway including *isg15, usp18* and *ube2l6*, (**Figures 1A–C**). Similarly, analysis of previously published transcriptomic datasets revealed increased expression of ISGylation-related genes in *M. tuberculosis*-infected human monocyte derived macrophages, and alveolar macrophages, as well as bone marrow-derived macrophages (**Supplementary Figure 1**).

The effect of IFN-I supplementation on ISGylation responses of *M. tuberculosis*-infected BMM was evaluated. Stimulation with IFN-β increased the expression of the ISGylation-related transcripts in uninfected BMM and further increased their levels in *M. tuberculosis*-infected cells. Transcripts encoding for *il1b, inos* and *tnf*, immune mediators that contribute to intracellular *M. tuberculosis* control, were increased in infected BMM (**Figures 1D–F**). The addition of IFN-β further enhanced the levels of these transcripts in infected, but not in uninfected, BMM.

In contrast to the stimulatory effects of IFN-β on ISGylation-related transcripts, stimulation of infected BMM with IFN-γ or IL-1β cytokines that improve control of *M. tuberculosis* infection (2, 23, 32, 33), did not further increase *isg15, usp18* and *ube2l6* mRNA levels beyond those induced by the infection alone (**Figures 1G–J, L-N**). Both IFN-γ and IL-1β increased levels of *inos* and *il1b* mRNA expression (**Figures 1K, O, P**).

### 3.2 Pharmacological inhibition of USP18 improves the intracellular control of infection with *M. tuberculosis*

**Figure 2:**
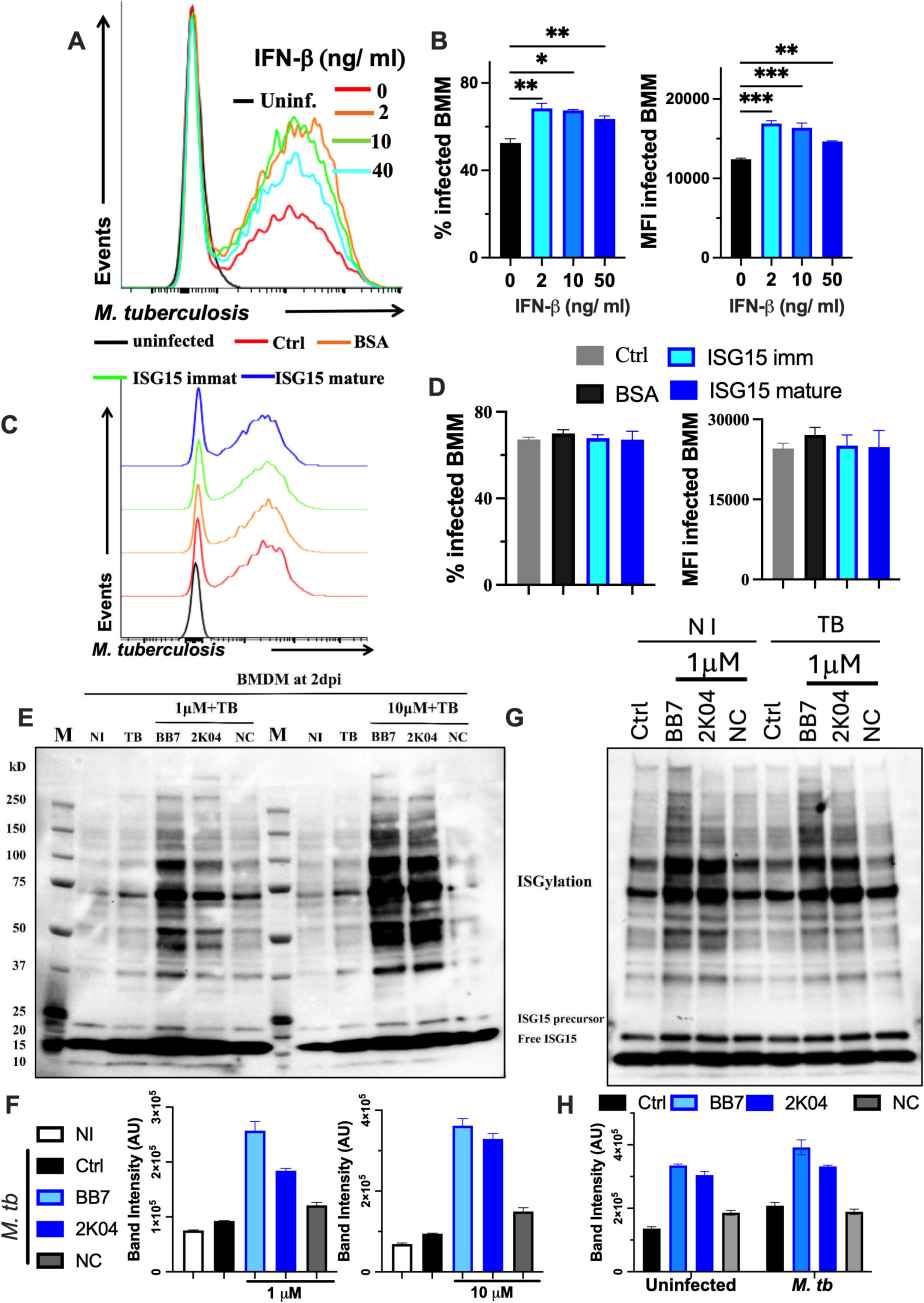
Small molecular inhibitors of USP18 enzymatic activity increase ISGylation in *M. tuberculosis-* infected and control BMM. **(A, B)** BMM were incubated with recombinant IFN-β at the indicated concentrations, and infected with *Mtb-gfp* 24h after the treatment. IFN-β was replenished 4h after infection. Representative histograms of *Mtb*-gfp-labelled BMM 5 days after infection, the % of infected BMM and the MFI of *Mtb* gfp in macrophages are shown. **(C, D)** Recombinant mature and capped mouse ISG15 were produced and incubated at 1μM with BMM 24h before infection with *M. tuberculosis*. BSA was used as a negative control. Representative histograms of *Mtb*-gfp-labelled BMM 5 days after infection, the % of infected BMM and the MFI of *Mtb* gfp in macrophages are shown. **(E–H)** BMM were treated with the indicated concentrations of BB7, 2K04 and NC (a negative control) 4h prior to infection with *M. tuberculosis***(E, F)**. Untreated non-infected (NI) controls were also added. The inhibitors and control compound (1 μM) were also incubated with *M. tuberculosis* infected and uninfected BMM **(G, H)**. BMM lysates were obtained 48h after *M. tuberculosis* infection and analyzed by Western blot using anti-ISG15 antibodies. Untreated (infected or uninfected cells in G are indicated as controls: Ctrl). The western blot bands from a representative sample per group **(E, G)** and the mean band intensity of 3 independent samples per group ± SEM **(F, H)** are shown. Differences are significant at *p<0.05, **p<0.01 and ***p<0.001, one-way ANOVA test.

Stimulation of BMM with various concentrations of IFN-β resulted in higher intracellular levels of *M. tuberculosis*, as measured by an increased frequency of infected BMM and a greater bacteria density per infected BMM (**Figures 2A, B**).

ISG15 is synthesized as 161 aminoacid-long precursor that is rapidly processed into its mature 155 aminoacid form by proteolytic cleavage, thereby exposing the conjugation site at its C-terminus (34). The immature (capped) ISG15 its not conjugated to substrates but may upon secretion, act extracellularly in a cytokine-like manner (12, 21). We produced both mature and capped ISG15 and tested whether these could influence *M. tuberculosis* control in BMM. The intracellular bacterial levels in BMM treated with different concentrations of mature or immature ISG15 were similar (**Figures 2C, D**).

Next, we investigated whether USP18 modulates macrophage responses to *M. tuberculosis* infection. For this purpose, two USP18 inhibitors, BB7 and 2K04, were used. These compounds were selected from a small-molecule compound library through a deISGylase assay-based high-throughput screening (2K04) and further optimized through medicinal chemistry (BB7) (28). BMM were treated with BB7 or 2K04 along with a structurally related inactive control compound (NC), before and during *M. tuberculosis* infection. Treatment of infected BMM with either 1 or 10 μM of BB7 or 2K04 but not with NC resulted in increased ISGylation (**Figures 2E, F**). Both inhibitors increased ISGylation in uninfected BMM (**Figures 2G, H**). *M. tuberculosis* infection resulted in increased ISGylation in BMM relative to uninfected controls (**Figures 2F, H**). In some experiments incubation with NC showed slightly increased ISGylation levels, although to a lesser extent than BB7 or 2K04 (**Figures 2F, H**). Incubation with the inhibitors did not reduce BMM viability (**Supplementary Figure 2A**), consistent with previous observations in cell lines at higher concentrations (28).

**Figure 3:**
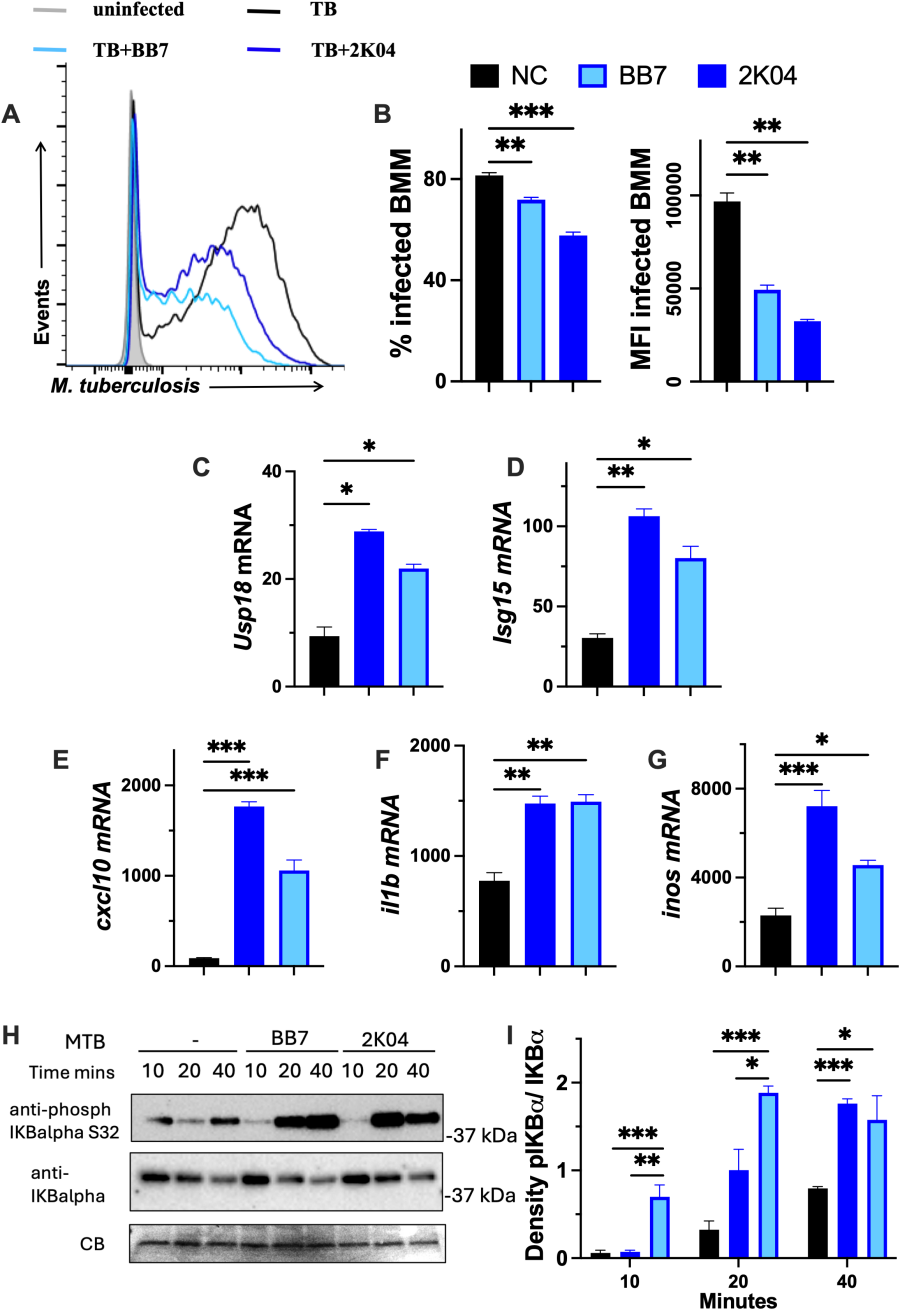
Inhibition of USP18 improves the intracellular control of infection with *M. tuberculosis.***(A, B)** BMM were incubated with either 10μM BB7, 2K04 or NC, 4 h before infection with *M. t* histograms of gfp content 5 days after infection, the percentage of infected BMM and the *Mtb-gfp* MFI of infected BMM at 5 days after infection are shown. **(C–G)** BMM were treated with either 10 μM BB7, 2K04 or NC 4 h before infection with *M. tuberculosis*. Total RNA was isolated 8 h after infection and the fold change of *isg15, usp18, cxcl10, il1b* and *inos* mRNA levels in comparison to a group of uninfected/untreated BMM were determined by RT-PCR. **(H, I)** BMM were treated with 10 μM BB7, 2K04 and NC (a negative control) 4h before infection with *M. tuberculosis*. Lysates were obtained at the indicated time points after infection and the expression of phospho IkΒα and total IkΒα was evaluated by Western Blot using specific antibodies. Commassie blue staining is shown as a loading control. The gel image **(H)** and the ratio of the band intensities of phospho- and total IkΒα for three independent samples pre group **(I)** are depicted. **(B–G)** The mean ± SEM of triplicate independent cultures is depicted. Differences are significant at *p ≤ 0.05, **p ≤ 0.01 and ***p ≤ 0.001, one-way ANOVA. Only differences with the negative control (NC) are shown.

We then tested whether USP18 inhibition by BB7 and 2K04 affected the control of *M. tuberculosis* infection in BMM. Cells treated with either BB7 or 2K04 showed improved control of intracellular *M. tuberculosis* when compared to BMM NC- treated controls, as indicated by the reduced frequency of infected BMM and the lower bacterial density per cell (**Figures 3A, B**). Coincubation of *M. tuberculosis* in axenic cultures with 10 μM USP18 inhibitors did not reduce the bacterial growth (**Supplementary Figure 2B**). Treatment of *M. tuberculosis*-infected BMM with either BB7 or 2K04 increased the levels of the IFN-I regulated *usp18, isg15* and *cxcl10* mRNA (**Figures 3C–E**), as well as *il1b* and *inos* (**Figures 3F, G**), which are transcriptionally regulated by NF-κB. The transcriptional activity of NF-κB is tightly repressed by binding to the IκBα inhibitor. Upon stimulation, IκBα is phosphorylated, leading to its the degradation and the release of NF-κB to activate gene transcription (35). Infection of BMM with *M. tuberculosis* increased IκBα phosphorylation, and treatment with the USP18 inhibitors further enhanced phospho-IκBα levels in infected BMM, demonstrating strong NF-κB activation by these inhibitors (**Figures 3H, I**).

### 3.3 Inflammasome activation and innate immune pathways are enriched in the transcriptome of *M. tuberculosis* infected BMM treated with USP18 inhibitor

**Figure 4:**
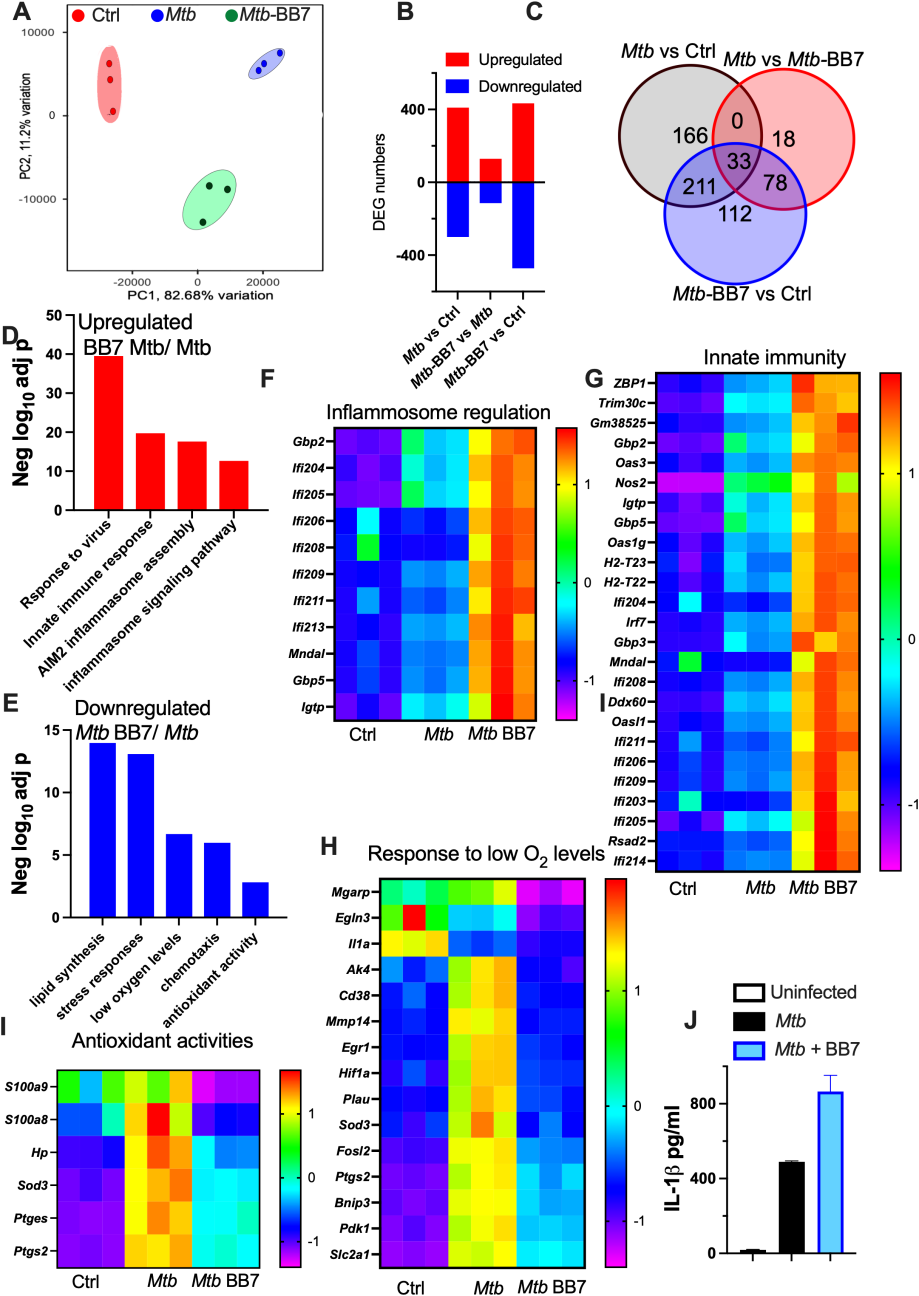
Inflammasome activation and innate immune pathways are enriched in the transcriptome of *M. tuberculosis* infected BMM treated with USP18 inhibitor. RNA seq was performed in triplicate independent cultures of BMM treated or not with either 10 μM BB7 or NC and infected 4 h after with *M. tuberculosis*. Untreated and uninfected macrophages were included as controls. RNA was extracted 8h after infection and RNA seq performed as indicated in Methods section. **(A)** PCA plots from non-supervised samples based on the normalized gene counts after filtering the low expressed genes are shown. **(B)** The number of differentially expressed genes (up or down-regulated) for each comparison combination is shown. **(C)** The Venn diagram represents the number of genes determine by RNA sequencing that are common to or uniquely differentially expressed within *Mtb*, *Mtb* -BB7, and uninfected controls. **(D, E)** The log_10_ p value of the most enriched terms after GO pathway analysis of *Mtb-BB7 vs Mtb* upregulated or downregulated DEGs were obtained by using the WebGestalt software. **(F–I)** The heat maps showing DEGs in the inflammosome **(F)**, innate immunity **(G)**, response to low O_2_ levels **(H)** and antioxidant **(I)** pathways were normalized by subtracting the log_2_ values to the mean log_2_ value for all samples for each gene in relation to the standard deviation of the gene determinations. **(J)** The titers of IL-1β in supernatants from BMM treated or not with BB7–4 h before infection with *M. tuberculosis* was evaluated by ELISA 72h after infection.

We next compared the transcriptomic profiles of *M. tuberculosis-*infected BMM treated with BB7 or NC. A differential clustering among uninfected, *M. tuberculosis* (*Mtb*) and *M. tuberculosis*-BB7-treated BMM was revealed by PCA analysis and Pearson’s correlation, highlighting the variation in gene expression between the diverse groups (**Figure 4A**, **Supplementary Figure 3A**). We identified 710 differentially expressed genes (DEGs) between uninfected and *Mtb* BMM and 243 DEGs comparing *Mtb-BB7 vs Mtb* BMM, 129 of these DEGs were upregulated (**Figures 4B, C**, **Supplementary Figure 3B**).

Gene ontology (GO) analysis revealed enrichment of pathways related to antiviral responses, innate immune activation and inflammasome signaling when comparing *Mtb-BB7* vs *Mtb* BMM (**Figure 4D**). Conversely, genes associated with stress responses, hypoxia and antioxidant activity were downregulated in *Mtb-BB7* (**Figure 4E**). A detailed analysis of individual transcripts within these pathways is shown in **Figures 4F–I** and **Supplementary Figures 3C, D**. ISGylation-related genes *usp18*, *ube2l6* and *isg15* were detected among transcripts of GO “responses to virus” that were upregulated by BB7 treatment. Additionally, the genes *inos, igtp, gbp2 and gbp5* which have been shown to contribute to the intracellular control of *M. tuberculosis* in macrophages (2, 36–38) were also increased in BB7-treated BMM (**Supplementary Figure 3C**). In contrast, expression of *arg1, socs3*, and *nr4a1* mRNA, which might impair anti-*M. tuberculosis* responses by macrophages was downregulated following BB7 treatment (**Supplementary Figure 3D**).

Because the inflammasome pathway was upregulated in BB7-treated, *M. tuberculosis*-infected BMM, mature IL-1β levels in culture supernatants were measured as a readout of inflammasome activation. IL-1β levels were stimulated by the *M. tuberculosis* infection but not by BB7 treatment alone. However, the addition of BB7 to *M. tuberculosis*-infected BMM increased IL-1β levels as compared to untreated controls, indicating that the USP18 inhibitor enhances the inflammasome pathway during infection (**Figure 4J**).

### 3.4 USP18 inhibition improves the control of *M. tuberculosis* by BMM in a IFN-I independent manner

**Figure 5:**
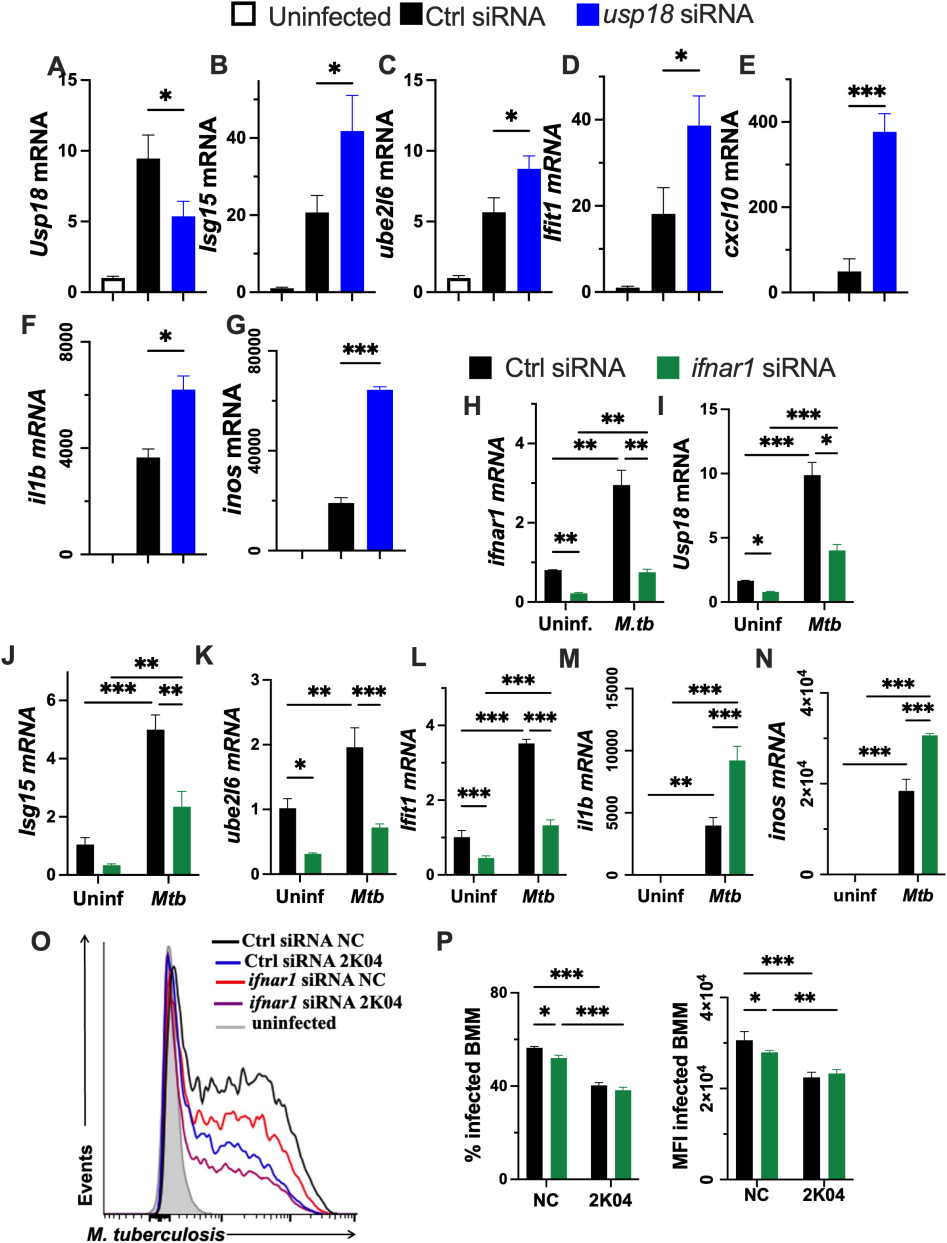
USP18 inhibitors improved the control of *M. tuberculosis* growth in BMM in a IFN-I-independent manner. **(A–G)** BMM were transfected with *usp18* or control siRNAs 24h before infection with *M. tuberculosis.* Total RNA was isolated 24 h after infection and the relative *usp18, isg15, ube2l6, ifit1, cxcl10, il1b* and *inos* mRNA levels were determined by RT-PCR. **(H–N)** BMM were transfected with *Ifnar1* or control siRNAs 24h before infection with *M. tuberculosis.* Total RNA was isolated 24 h after infection and the relative *ifnar1*, *isg15, usp18, ube2l6, ifit1, il1b* and *inos* mRNA levels were determined by RT-PCR. **(O, P)** BMM were transfected with either *Ifnar1* or control siRNAs 24h before treatment with either small molecule USP18 inhibitor 2K04 or NC. BMM were infected with *M. tuberculosis*-gfp 4h after treatment. Representative histograms of *M. tuberculosis*-gfp levels **(O)**, the percentage of infected BMM and the *M. tuberculosis-gfp* MFI of infected BMM at 5 days after infection **(P)** are shown. **(A–N, P)** The mean ± SEM of triplicate independent cultures is depicted. Differences are significant at *p ≤ 0.05, **p ≤ 0.01 and ***p ≤ 0.001, one-way **(A–H)** and two-way ANOVA **(H–N, P)**.

Silencing of USP18 by siRNA transfection, reduced the accumulation of *usp18* mRNA in *M. tuberculosis*-infected BMM (**Figure 5A**). Conversely, levels of the ISGylation related transcript *isg15* and *ube2l6*, as well as the IFN-I regulated genes *ifit1* and *cxcl10*, were increased following *usp18* silencing (**Figures 5B–E**). Similarly, *usp18*-silenced infected BMM displayed increased *il1b* and *inos* mRNA levels (**Figures 5F, G**), recapitulating the effect of pharmacological USP18 inhibition.

Then, we investigated whether IFN-I signaling contributes to the role of USP18 in *M. tuberculosis* infection of BMM. Silencing *ifnar1*, which encodes the IFN-I receptor α chain, reduced the accumulation of *ifnar1* mRNA (**Figure 5H**) and decreased the expression of IFN-I regulated genes *usp18, isg15, ube2l6* and *ifit1* as compared with control siRNA-transfected BMM (**Figures 5I–L**), indicating that the IFN-I signal was hampered by the *ifnar1* siRNA. Levels of *il1b* and *inos* mRNA were increased in *ifnar1* siRNA transfected BMM as compared to those in BMM transfected with control siRNA (**Figures 5M, N**).

Next, we examinate whether *ifn1ar* silencing hindered the ability of USP18 inhibitors to control *M. tuberculosis*. Treatment with 2K04 reduced both the proportion of infected BMM and bacterial density per infected cell compared with controls (**Figures 5O, P**). Blocking IFN-I signaling through *ifna1r* siRNAs similarly decreased the intracellular bacterial load, and combining 2K04 treatment with *ifna1r* silencing did not alter the enhanced *M. tuberculosis* control mediated USP18 inhibition (**Figures 5O, P**). These results indicated that the improved control of the intracellular bacteria after USP18 inhibition is independent of IFN-I signaling.

**Figure 6:**
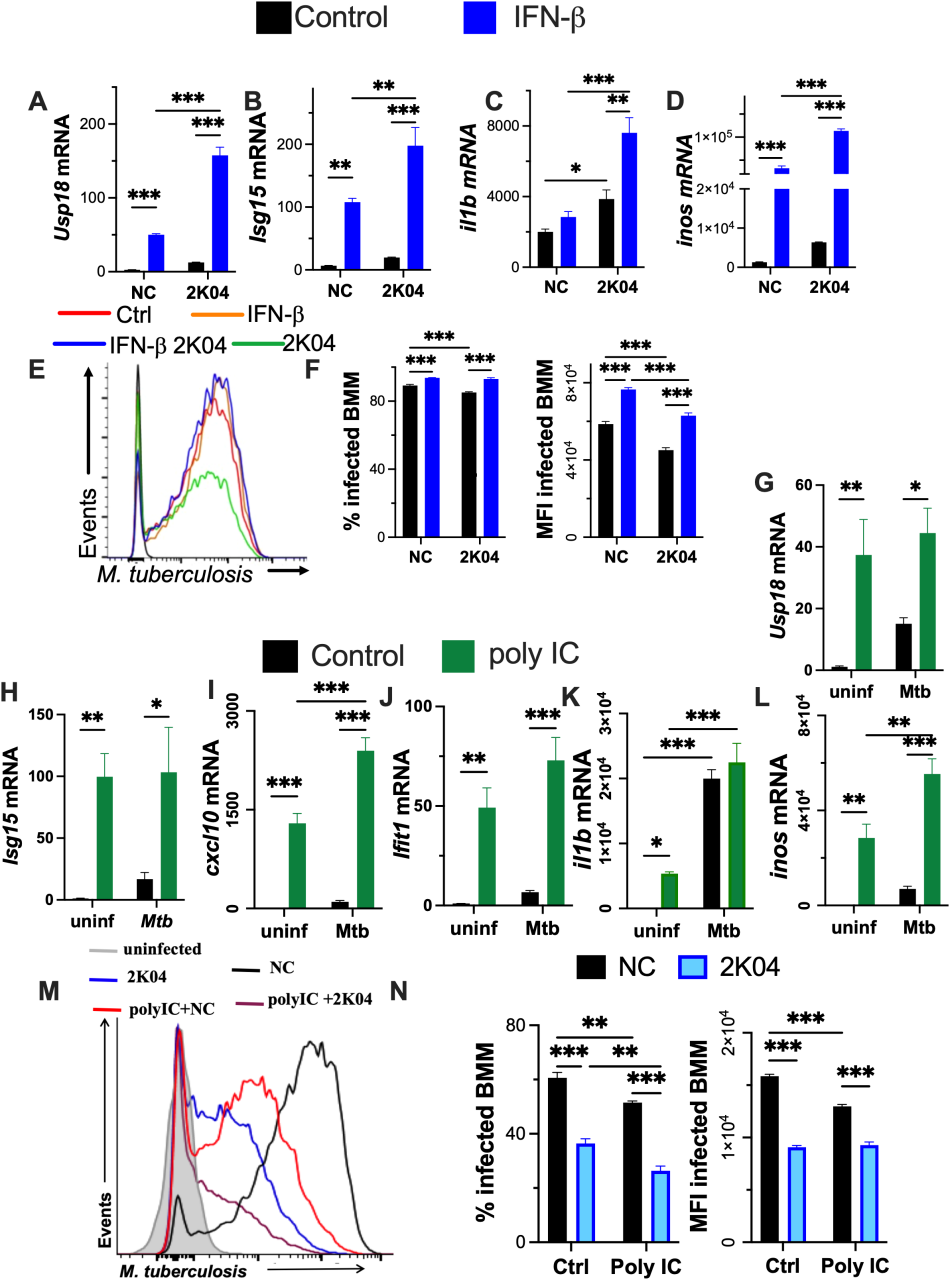
IFN-β supplementation hampers the control of *M. tuberculosis* infection in USP18 inhibitor-treated BMM. **(A–D)** BMM were stimulated with IFN-β, treated with either the usp18 inhibitor 2K04 or NC 24 h after and 4h after the treatment with the inhibitor infected with *M. tuberculosis*. Total RNA was isolated from BMM 24h after infection and the relative levels of *usp18, isg15, il1b* and *inos* mRNA determined by RT-PCR. **(E, F)** BMM were incubated with IFN-β, treated 24h after with either 2K04 or NC and 4h after the treatment with the USP18 inhibitor infected with *M. tuberculosis*-gfp. The extracellular bacteria was washed 4h after infection and the inhibitor replenished. Representative histograms of *M. tuberculosis*-gfp **(G)**, the % of infected BMM and the *M. tuberculosis-gfp* MFI of infected BMM at 5 days after infection **(H)** are shown. **(G–L)** BMM were treated with 5 μg/ml polyIC 24h before infection with *M. tuberculosis*. Total RNA was isolated 24 h after infection and the fold change of *isg15, usp18, cxcl10, ifit1, il1b* and inos mRNA were quantified by RT-PCR. **(M, N)** BMM were treated with 5 μg/ml polyIC and 24 h after incubated with either 10 μM 2K04 or NC. BMM were infected with *M. tuberculosis*-gfp for 4h when extracellular bacteria was washed and inhibitors replenished. Representative histograms of *M. tuberculosis*-gfp **(M)**, the % of infected BMM and the *M. tuberculosis-gfp* MFI of infected BMM at 5 days after infection **(N)** are shown. **(A–D, F–L, N)** The mean ± SEM of triplicate independent cultures is depicted. Differences are significant at *p ≤ 0.05, **p ≤ 0.01 and ***p ≤ 0.001, two-way ANOVA.

The effect of IFN-β supplementation in combination with USP18 inhibition in the responses of *M. tuberculosis*-infected BMM was then evaluated. The levels of *isg15* and *usp18* mRNA in *M. tuberculosis*-infected BMM increased after the stimulation with IFN-β. These effects were further amplified upon IFN-β and 2K04 co-treatment (**Figures 6A, B**). Levels of *il1b* and *inos* transcripts were also higher in IFN-β-treated *M. tuberculosis*-infected BMM exposed to 2K04 than in NC-treated controls (**Figures 6C, D**). However, IFN-β stimulation partially impaired the improved bacterial control conferred by the USP18 inhibitor (**Figures 6E, F**). Thus, high levels of IFN-I combined increased the susceptibility to *M. tuberculosis* in BMM with impaired USP18 regulation.

Finally, we assessed whether stimulation with the TLR3 agonist polyIC, a potent inducer of IFN-I expression, would mimic the effects of IFN-β addition on USP18 inhibitor-treated BMM. PolyIC induced the accumulation *isg15, usp18, ifit1* and *cxcl10* transcripts in both uninfected and *M. tuberculosis*-infected BMM (**Figures 6G–K**). PolyIC also increased the levels of *il1b* and *inos* mRNA in both uninfected and infected BMM (**Figures 6K, L**), while IFN-β-induced the accumulation of *i1b* and *inos* mRNA in infected but not in uninfected BMM (**Figures 1D–F**). Moreover, the addition of polyIC reduced the intracellular density of *M. tuberculosis* as compared to untreated controls (**Figures 6M, N**). Combined treatment with both polyIC and 2K04 further decreased the intracellular bacterial levels in BMM relative to either treatment alone (**Figures 6M, N**).

## 4 Discussion

Here we shown that infection of BMM with *M. tuberculosis* stimulates the expression of genes involved in the ISGylation pathway. ISGylation-related transcripts were also increased in human and murine inflammatory and alveolar macrophages. Studies indicating that transcript and protein levels of ISGylation components in blood may serve as biomarkers for TB diagnosis further support our findings (39–41).

The addition of IFN-β stimulated the expression of ISGylation-related transcripts in uninfected BMM and enhanced their expression in *M. tuberculosis*-infected BMM. Conversely, ISGylation transcript levels in *M. tuberculosis*-infected BMM with defective IFN-I-signaling were reduced. The stimulation of BMM with either IFN-γ or IL-1β did not modulate the concentration of ISGylation-related transcripts. Interestingly, the addition of IFN-I increased the of *il1b* and *inos* mRNA in *M. tuberculosis*-infected-, but not in uninfected BMM. Paradoxically, silencing of IFN-I signaling also resulted in increased *il1b* and *inos* expression in BMM.

In addition to functioning as a protein modifier, ISG15 has also been shown to act extracellularly by stimulating IFN-γ secretion through binding to the LFA-1 surface receptor (12, 21). We found that the addition of ISG15 to the BMM cultures did not affect the intracellular *M. tuberculosis* growth. However, the possibility that ISG15 may act indirectly, through interactions between different immune cell populations, to influence the intracellular replication of *M. tuberculosis* in macrophages remains to be explored.

USP18 dampens IFN-I signaling preventing IFN-I-mediated inflammation that is observed in patients with USP18 deficiency (18, 19, 42). The inhibitory effect of USP18 on IFN-I signaling is independent of its isopeptidase activity, since control of IFN-I responses is preserved in mice with catalytically inactive USP18 (17, 43). This function is also independent of ISG15, as demonstrated in *usp18^-/-/^isg15^-/-^* mice (44). Mechanistically, USP18 interacts with STAT2, facilitating its recruitment to the IFNAR2 subunit of the IFN-I receptor and inhibiting receptor dimerization (45, 46). Distinct regions of USP18 mediate deISGylation and inhibition of IFN-I signaling (17). The inhibitors BB7 and 2K04 used in this study were selected based on their ability to covalently bind USP18 and impair its enzymatic activity to cleave ISG15-protein substrates (28). Here, we demonstrate that these inhibitors also hamper the negative regulatory function of USP18 on the IFN-I signaling.

Although the role of ISG15 in our model has not been elucidated, ISG15 is required for rescuing USP18 from S‐phase kinase‐associated protein 2‐mediated proteasomal degradation, a mechanism that explains the interferonopathies and increased susceptibility to environmental mycobacterial infections observed in patients with ISG15 deficiencies (22, 47). In contrast, *isg15-/- or ube1l*^−/−^ mice did not show higher susceptibility to *M. tuberculosis* infection (48, 49), likely because the stabilizing effect of ISG15 on USP18 is not conserved in mice (20).

Patients with active tuberculosis have a characteristic IFN-I-inducible gene signature (4). While inhibition of USP18 increases the IFN-I responses in BMM, we here showed that the USP18 inhibition impairs the intracellular growth of *M. tuberculosis* in BMM in an IFN-I-independent manner.

*Usp18^-/-^ and usp18Ity9* mice, the latter with a mutation on USP18 that disrupts the IFN-I inhibitory function but not its enzymatic activity, are more susceptible to Salmonella infection (48, 50, 51). Similarly, *usp18Ity9* also showed increased *M. tuberculosis* susceptibility. In contrast, *usp18* and *ifnar2-*deficient mice showed improved control of *L. monocytogenes* (52).

Previous studies have provided evidence that USP18 can inhibit NF-κB activation by promoting deubiquitination of the TAK1–TAB1 complex or NEMO (53, 54). However, USP18 exhibits no reactivity toward ubiquitin *in vitro*, suggesting that the inhibition of the NF-κB pathway is indirect (17, 43). USP18-mediated NF-κB inhibition also protected mice from LPS-induced sepsis (55). Consistent with this, we showed that the inhibition of USP18 increased the expression of both IFN-I mediated but also of NF-κB-controlled inflammatory genes that have been shown to mediate the control of *M. tuberculosis* growth in macrophages. In agreement with the observed enrichment of inflammasome activation pathways, mature IL-1β was also secreted to the supernatants of *M. tuberculosis* infected BMM treated with the USP18 inhibitors. Similarly with our results, IL-1β is increased at both the transcript and protein levels in *usp18^-/-^* mice infected with Salmonella (48).

While being a potent IFN-I activator, polyIC has also been shown to also stimulate the JNK, p38 kinases and NF-kB mediated signaling pathways (56, 57). Accordingly, we found that stimulation with polyIC induced both IFN-I and inflammatory responses, reduced the *M. tuberculosis* levels in BMM and that co-incubation with both polyIC and a USP18 inhibitor further improved *M. tuberculosis* intracellular control.

Thus, while IFN-I responses are increased in USP18 deficient macrophages, an IFN-I independent inhibition of *M. tuberculosis* growth associated to the activation of NF-κB dependent immune molecules prevailed. Supporting this conclusion, C57BL/6 mice, used in this study, mount relatively weak IFN-I responses to *M. tuberculosis*, and deletion of *ifnar1* in this background does not consistently alter pulmonary bacterial burden (58–60). It is also possible that additional IFN-I producing cells, such as plasmacytoid DCs are required to potentiate the detrimental role of IFN-I during *in vivo M. tuberculosis* infections (5). Although our findings may appear to contrast with previous reports showing that IFN-I limits pro–IL-1β availability and IL-1β maturation (7) and that elevated IFN-I inhibits *M. tuberculosis*-induced IL-1β mRNA expression in macrophages (61), the addition of IFN-β to *M. tuberculosis* inhibited the protective effect of the USP18 inhibitors on the control of the bacterial growth in macrophages.

**Figure 7:**
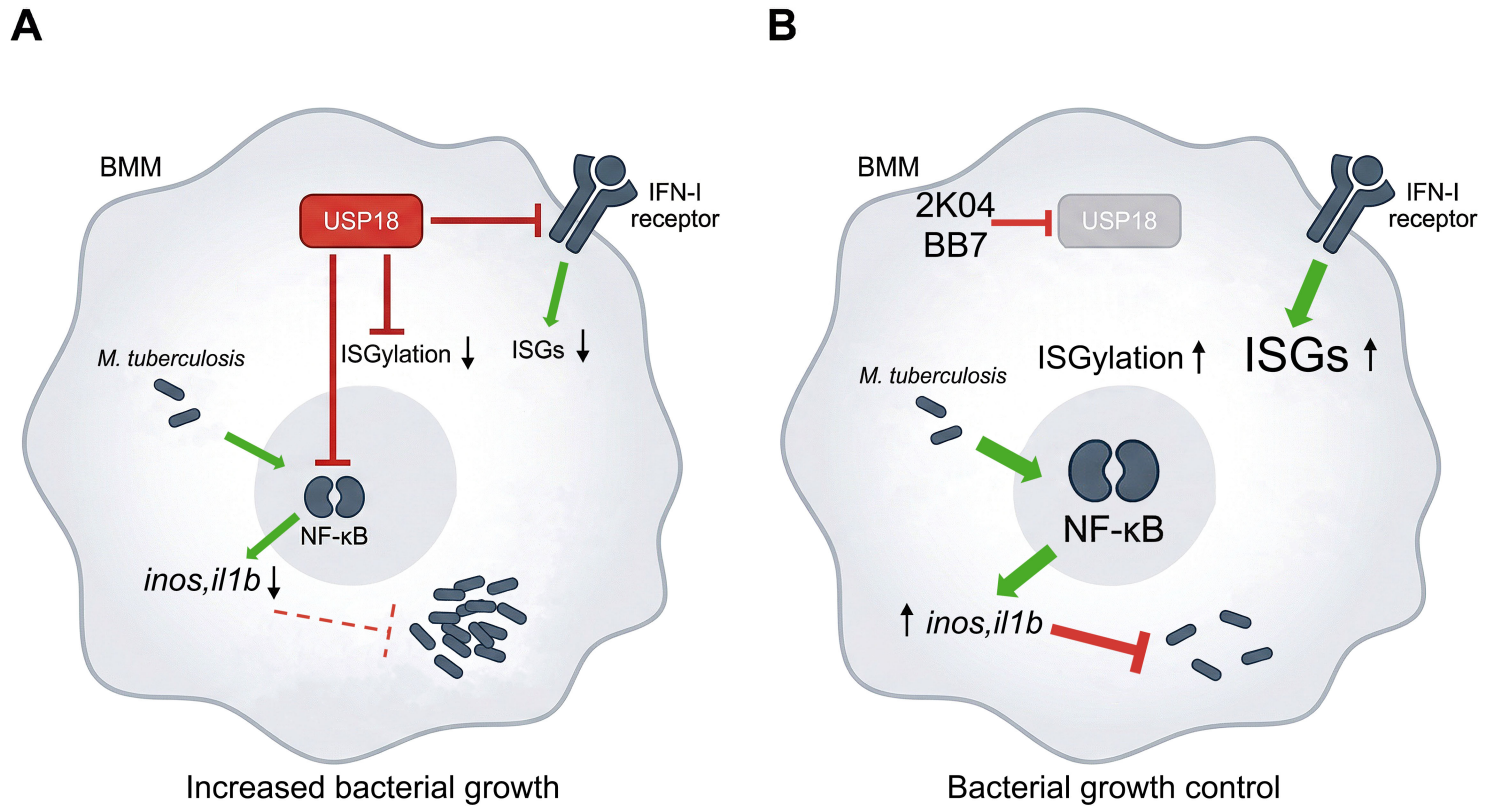
Graphical summary. The infection of BMM with *M. tuberculosis* in macrophages stimulates the expression of IFN-I dependent genes involved in the ISGylation pathway, including USP18. During *M. tuberculosis* infection USP18 negatively regulates ISGylation, IFN-I responses and the activation of the NF-κB pathway **(A)**. The inhibitors BB7 and 2K04 selected based on their ability to covalently bind to USP18 impairing its enzymatic activity to cleave ISG15-protein, increased ISGylation when incubated with BMM. The inhibitors also hampered the negative regulatory function of USP18 on IFN- and on NF-κB signaling resulting in that elevated the expression of genes that mediate the control of *M. tuberculosis* growth. We show that while IFN-I responses are increased in USP18 deficient macrophages, an IFN-I independent inhibition of *M. tuberculosis* growth associated to the activation of NF-κB dependent immune molecules prevails **(B)**.

Overall, our findings using small-molecule inhibitors that disrupt both the deISGylating and scaffold functions of USP18, reveal that USP18 limits the full potential of BMM to control the *M. tuberculosis* infection in an IFN-I independent manner (**Figure 7**).

## Data Availability

The data presented in the study are deposited in the GEO repository, accession number GSE320445.
