# Supplementary material for: Pharmacological inhibition of USP18 improves antibacterial responses and the intracellular control of Mycobacterium tuberculosis in macrophages
Source: Front Immunol. 2026 Mar 4;17:1739628. doi: 10.3389/fimmu.2026.1739628 (PMC12996095; doi:10.3389/fimmu.2026.1739628)
Supplement: Supplementary file 1 [file DataSheet1.pdf]

Supplementary Material

1 Supplementary Figures and Tables

1.1 Supplementary Figures

Supplementary Figure 1

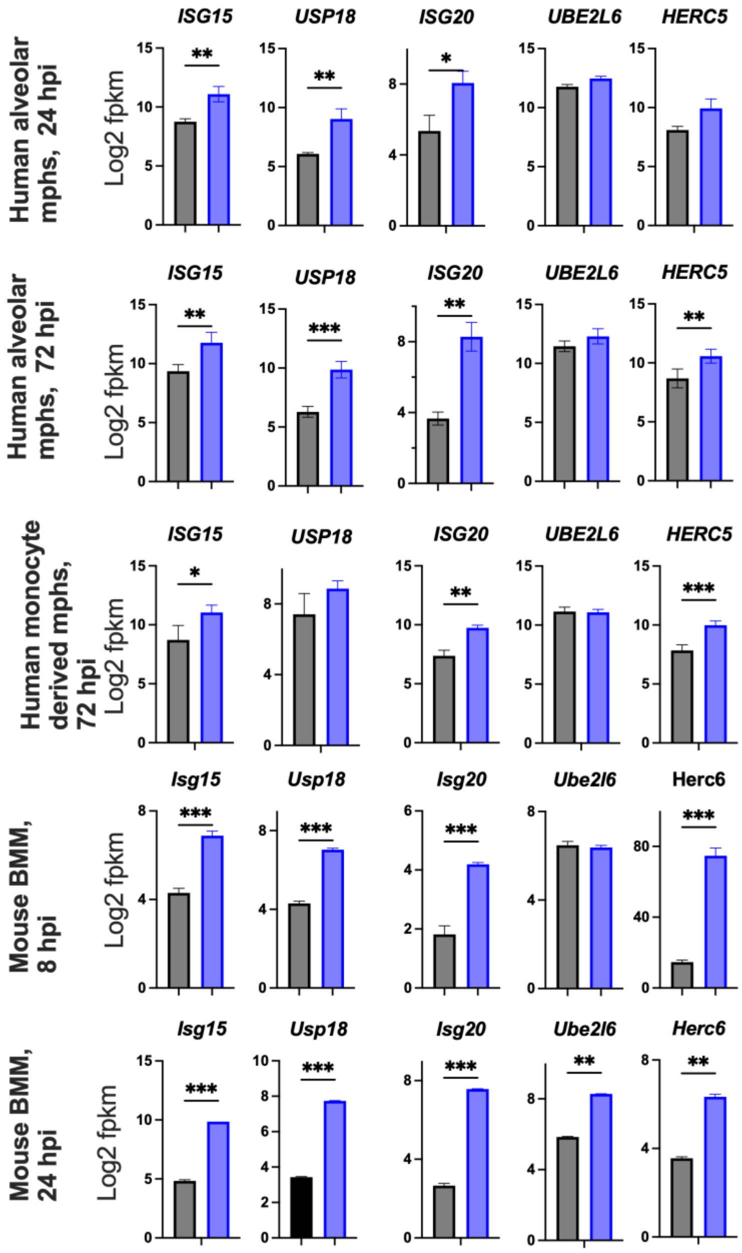

Increased expression of ISGylation-related transcripts in different human and mouse macrophages after infection with M. tuberculosis

Previously published macrophage gene expression datasets were downloaded from Gene Expression Omnibus. (A) GSE189996 dataset of human alveolar macrophages 24hpi (n=5 per group) [1]; GSE223863 human alveolar macrophages 72 hpi, (n=5 per group) [1] (B); GSE164287 human monocyte derived macrophages 48 hpi (n=5 per group) [2] (C), GSE271061 mouse bone marrow macrophages 8 hpi (n=3 per group) [3](D); and GSE289356 mouse bone marrow macrophages 24 hpi (n=3 per group)[4] (E) were mined for the analysis. In all cases comparison between uninfected and Mtb infected macrophages is shown. The mean $\log_2$ fpkm ± SEM of selected transcripts coding for ISGylation related proteins is shown. Differences are significant at *p≤0.05, **p≤0.01 and ***p≤0.001, unpaired Student's *t* test with Welch correction.

References to the data sets used

1. Sadee W, Cheeseman IH, Papp A, Pietrzak M, Seweryn M, Zhou X, et al. Human alveolar macrophage response to Mycobacterium tuberculosis: immune characteristics underlying large inter-individual variability. Commun Biol. 2025;8(1):950.

2. Looney M, Lorenc R, Halushka MK, Karakousis PC. Key Macrophage Responses to Infection With Mycobacterium tuberculosis Are Co-Regulated by microRNAs and DNA Methylation. Front Immunol. 2021;12:685237.

3. Li H, Liu R, Kathamuthu GR, Gencheva R, Gong Z, Scholz AT, et al. The inhibition of TXNRD1 by methylglyoxal impairs the intracellular control of Mycobacterium tuberculosis. Redox Biol. 2025;85:103741.

4. Yang Z, Zhang L, Ottavi S, Geri JB, Perkowski A, Jiang X, et al. ACOD1-mediated lysosomal membrane permeabilization contributes to Mycobacterium tuberculosis-induced macrophage death. Proc Natl Acad Sci U S A. 2025;122(12):e2425309122.

Supplementary Figure 2

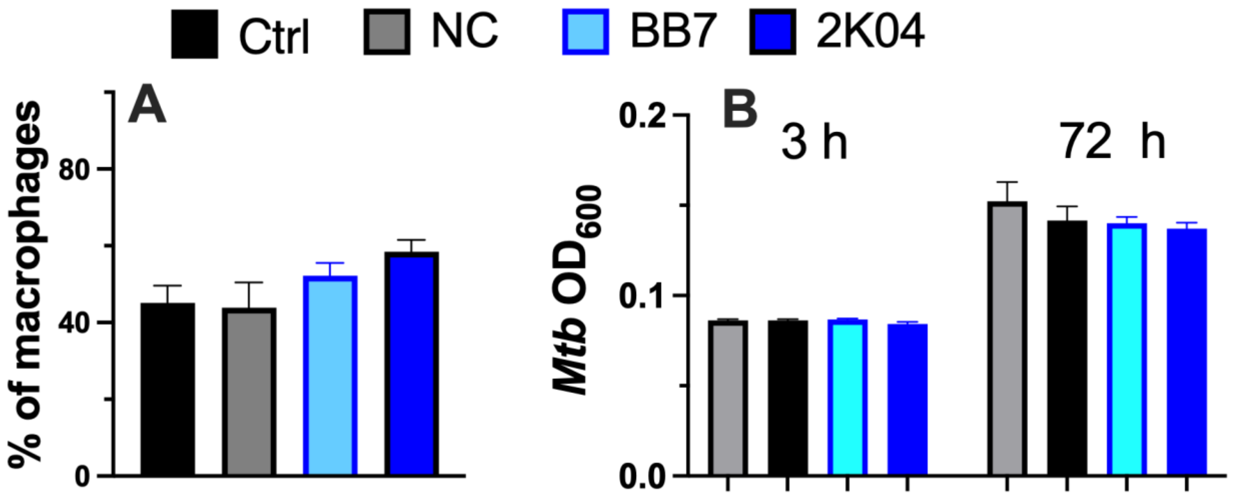

The USP18 inhibitors hampered neither the viability of BMM nor the growth of *M. tuberculosis* in axenic cultures.

(A) BMM were incubated with 10 μM BB7, 2K04 or NC 4h before infection with *M. tuberculosis*. The % of events in the macrophage gate (FCS/SSC) were measured 5 days after infection. The mean % macrophages ± SD of triplicate independent cultures per group is shown **(B)** *M. tuberculosis* $3x10^6$ were cultured Middlebrook 7H9 (Difco, Detroit, MI) supplemented with albumin, dextrose and catalase were co-incubated with 10 μM BB7, 2K04 or NC at 37°C. *M. tuberculosis* were quantified by densitometry 3h and 72h after. The mean $OD_{600\ nm}$ ± SD of triplicate cultures per condition is shown.

Supplementary Figure 3

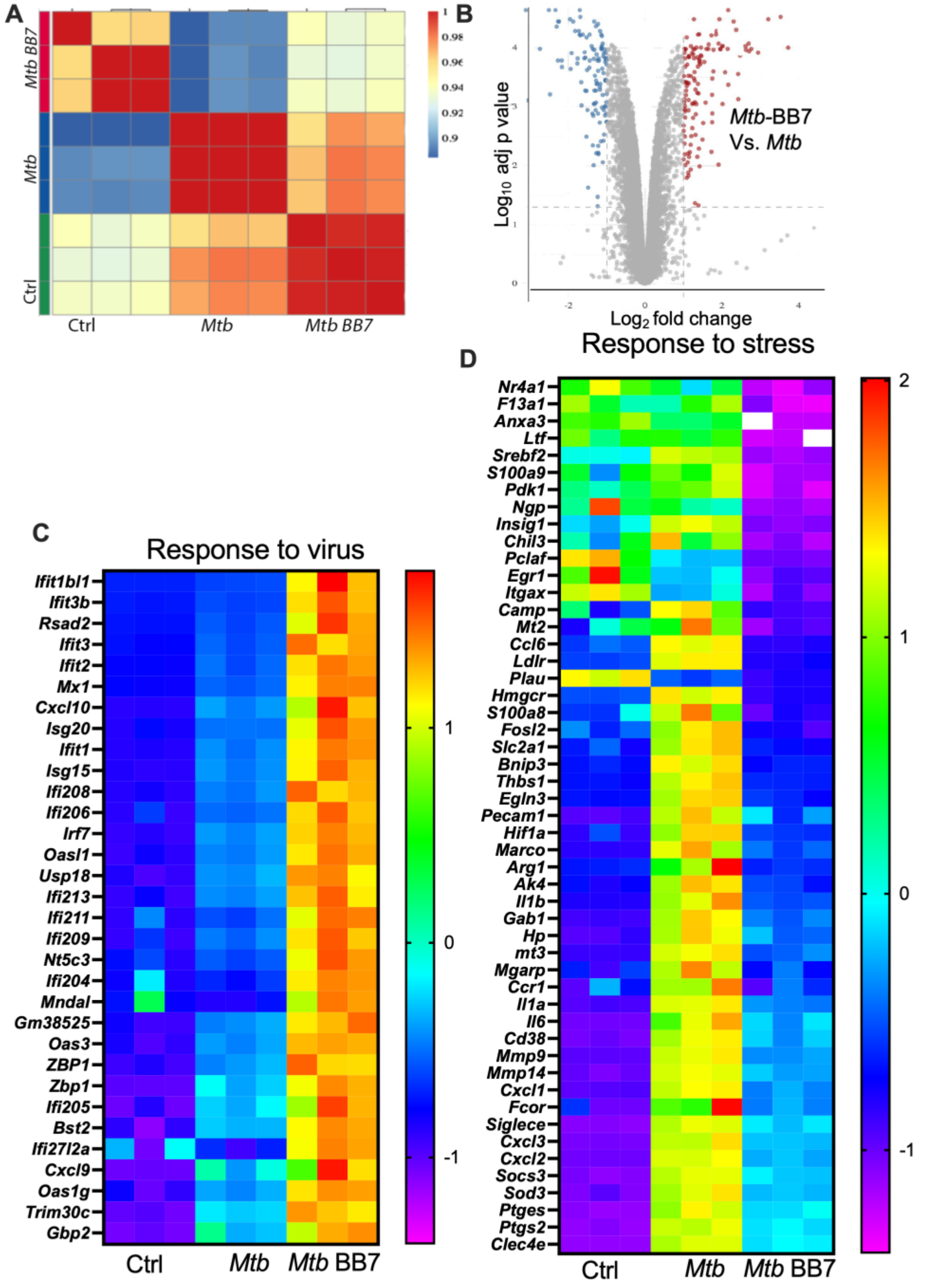

Differential gene expression after treatment with BB7 of M. tuberculosis-infected BMM

(A) RNA seq was performed in triplicate independent cultures of BMM treated or not with 10 µM BB7 and infected 4 h after with *M. tuberculosis*. The total RNA was extracted 8h after infection and sequencing was performed as indicated in Methods section. The Pearson R^2 correlation coefficient matrix of the expression level of all genes in each sample is shown as a heat map. **(B)** Volcano plots

showing the log_2 fold change (the gene expression difference between both samples) in the x-axis and the statistical significance in the y-axis, representing the overall distribution of differentially upregulated or downregulated genes in uninfected vs. *Mtb*-infected and *Mtb* vs *Mtb*-BB7 BMM are depicted. **(C and D)** The heat maps showing DEGs in the response to virus and response to stress pathways were normalized by subtracting the log_2 values to the mean log_2 value for all samples for each gene in relation to the standard deviation of the gene determinations.

Supplementary Figure 4

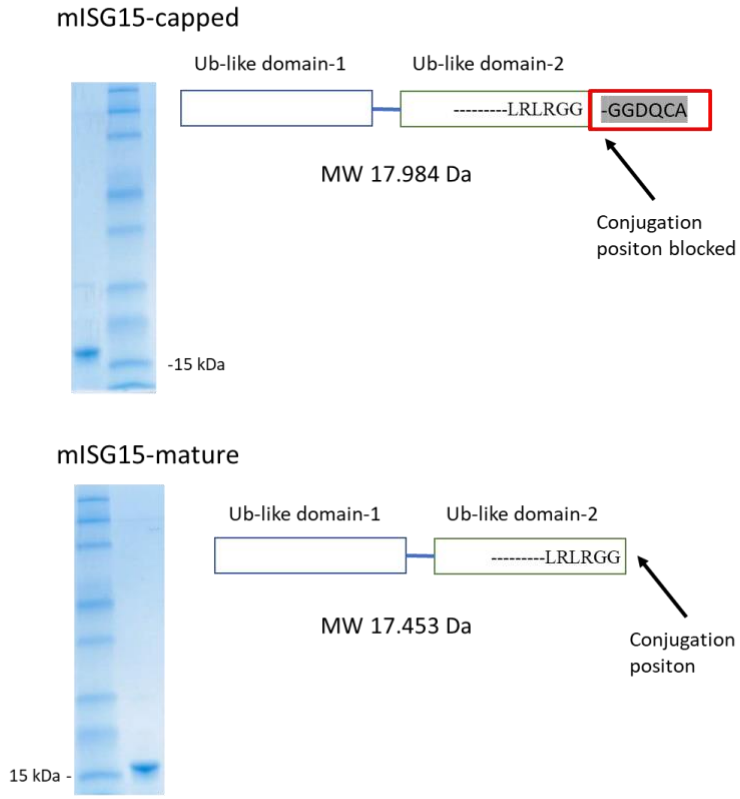

Preparation of recombinant ISG15

Mouse ISG15 (mISG15) was expressed in E.coli and purified to homogeneity. The two constructs represent the i.) capped pre-protein (top panel) carrying the C-terminal hexapeptide tag (`156-GGDQCA-161`) that blocks the conjugation site and the ii.) mature ISG15 (lower panel) lacking this sequence element where the conjugation site is accessible. SDS-PAGE analysis of the purified proteins is shown for both constructs.

1.2. Supplementary Tables

Supplementary table 1

Sequences of RT-PCR primers

Targets	Forward	Reverse
hprt	CCCAGCGTCGTGATTAGC	GGAATAAACACTTTTTCCAAATCC
isg15	GGTGTCCGTGACTAACTCCAT	CTGTACCACTAGCATCACTGTG
ube2l6	GAGTGGCGAAAGAGCTGGAG	TTGGCATCGTCACTAGACAGT
usp18	TGCCTCGGAGTGCAGAAGA	CGTGATCTGGTCCTTAGTCAGG
il1b	TGGTGTGTGACGTTCCCATT	CAGCACGAGGCTTTTTTGTTG
inos	CAGCTGGGCTGTACAAACCTT	CATTGGAAGTGAAGCGTTTCG
tnfa	CAGGCGGTGCCTATGTCTC	CGATCACCCCGAAGTTCAGTAG
ifit1	GCCTATCGCCAAGATTTAGATGA	TTCTGGATTTAACCGGACAGC
cxcl10	CCAAGTGCTGCCGTCATTTTC	GGCTCGCAGGGATGATTTCAA
ifnar1	GACAACTACACCCTAAAGTGGAG	GCTCTGACACGAAACTGTGTTTT

Supplementary table 2

Sequences of si RNA

	Forward	Reverse
usp18	GGAAUCCCGUGGAUGGAAATT	UUUCCAUCCACGGGAUUCCGG
ifnar1	GGAAUGAGGUUGAUCCGUUTT	AACGGAUCAACCUCAUUCCAC
